# Dexamethasone‐Induced Avascular Necrosis of the Femoral Head in a Patient With Major Depressive Disorder: A Rare Case Report

**DOI:** 10.1002/ccr3.71591

**Published:** 2025-12-07

**Authors:** Reza Bidaki, Asma Khosravi Najafabadi, Farzaneh Hasani

**Affiliations:** ^1^ Department of Psychiatry, Research Center of Addiction and Behavioral Sciences, Non‐Communicable Diseases Research Institute Shahid Sadoughi University of Medical Sciences Yazd Iran; ^2^ Student Research Committee Shahid Sadoughi University of Medical Sciences Yazd Iran

**Keywords:** depression, depressive disorder, femur head necrosis, osteonecrosis

## Abstract

Avascular necrosis (AVN) of the femoral head, also known as osteonecrosis, is one of the main causes of disability during the most productive years of life and results in significant financial consequences. This condition most commonly occurs in patients with long‐term and inappropriate corticosteroid use, particularly dexamethasone. This is a rare case report of AVN and steroid‐induced depression in a patient who was wrongly advised by peers to administer dexamethasone to counteract the physical decline associated with chronic substance use. This case highlights the necessity of regular screening for corticosteroid misuse in patients with chronic substance use and highlights the need to develop preventive approaches.

**Summary**

- Depression and avascular necrosis (AVN) are two potential side effects of long‐term dexamethasone use to counteract physical decline in a patient with chronic opium use. This case illustrates the link between unprescribed corticosteroid use, particularly dexamethasone, and the development of avascular necrosis (AVN), highlighting the importance of a preventive approach.

## 1 Introduction

Corticosteroids are commonly used in the treatment of a variety of ailments, particularly as anti‐inflammatory agents, making them widely used in the community. Despite their life‐saving effects, unsupervised and long‐term use can cause severe adverse effects, such as weight gain due to visceral fat accumulation, muscle and bone loss, including avascular necrosis (AVN), and mental disorders such as depression [1].

AVN is a clinical and debilitating condition caused by insufficient blood flow to the femur, leading to progressive and vitiating changes in the femoral head and hip joint, which are the most commonly involved regions. A wide etiological spectrum has been considered for AVN, including long‐term corticosteroid use, notably dexamethasone, at the top of the list [2].

Between 20,000 and 30,000 new cases of AVN are estimated to be diagnosed annually in the USA, which usually affects young individuals and causes lifelong disability if it remains untreated [3].

Additionally, corticosteroids are ambiguous drugs that initially induce euphoria and reduce anxiety, which increases the willingness to misuse and may lead to depression in the long term [4]. The following report presents a previously unreported case of chronic multi‐substance use that was unsuccessful in finding a solution to overcome opium‐induced physical changes, including facial atrophy and weight loss, by using dexamethasone.

## 2 Case History/Examination

A 30‐year‐old unmarried male, previously employed in manual labor and construction, with formal education discontinued at age 15, presented to the outpatient clinic with complaints of persistent fatigue and generalized weakness that began following cessation of opioid use. He had a prior history of hospitalization for substance use and was subsequently admitted to the psychiatric ward for comprehensive evaluation and management.

He reported a persistently depressed mood accompanied by verbal and physical aggression, anxiety, and impaired social and occupational functioning. Despite sleeping approximately 9 h per night, he experienced chronic fatigue and a lack of motivation. He exhibited reduced verbal communication and spent the majority of his time at home, remaining inactive. Occasionally, he socialized with friends and used marijuana during these encounters. He did not express hopelessness or suicidal ideation but rather labeled guilt over past behaviors. Notably, he also reported an increased libido during the current episode.

No significant evidence of perceptual disturbances, such as auditory or visual hallucinations, was observed. The patient also had no delusions, ideas of reference, or symptoms suggestive of obsessive‐compulsive disorder.

He had a 12‐year history of chronic opium use. He began misusing dexamethasone approximately 2 years after initiating opioids, that is about 10 years ago, reportedly to mitigate the physical consequences of opioid use, including weight loss, facial atrophy, and hyperpigmentation, and to enhance physical performance. The patient had been using dexamethasone irregularly and without a medical prescription, sometimes omitting doses or taking them at lower levels, and at other times consuming higher doses. Over time, he developed symptoms of depression, irritability, anxiety, and restlessness, leading to the use of clonazepam and trihexyphenidyl to self‐medicate. He also reported the past use of tramadol.

He was admitted to drug rehabilitation centers approximately 15 times and fulfilled the diagnostic criteria for major depressive disorder (MDD). There was no history of psychosis, delirium, or mania. His longest abstinence period lasted about 8 months; his most recent admission occurred 2 years ago and lasted only 2 weeks. During psychiatric treatment, he received electroconvulsive therapy (ECT) and responded favorably to pharmacological interventions.

At the time of the current admission, he reported daily cigarette use (one pack/day), methadone (80 mg/day), cyproheptadine, and cannabis. He also mentioned taking aspirin, apixaban, and meloxicam due to orthopedic surgery. His prior substance use included alcohol (last use: 2 years ago), buprenorphine (last use: 1 year ago), tramadol (last use: 8 years ago), and dexamethasone, which he discontinued 2 months prior to the current admission to the psychiatric clinic.

There was no family history of psychiatric disorders or substance use.

**FIGURE 1:**
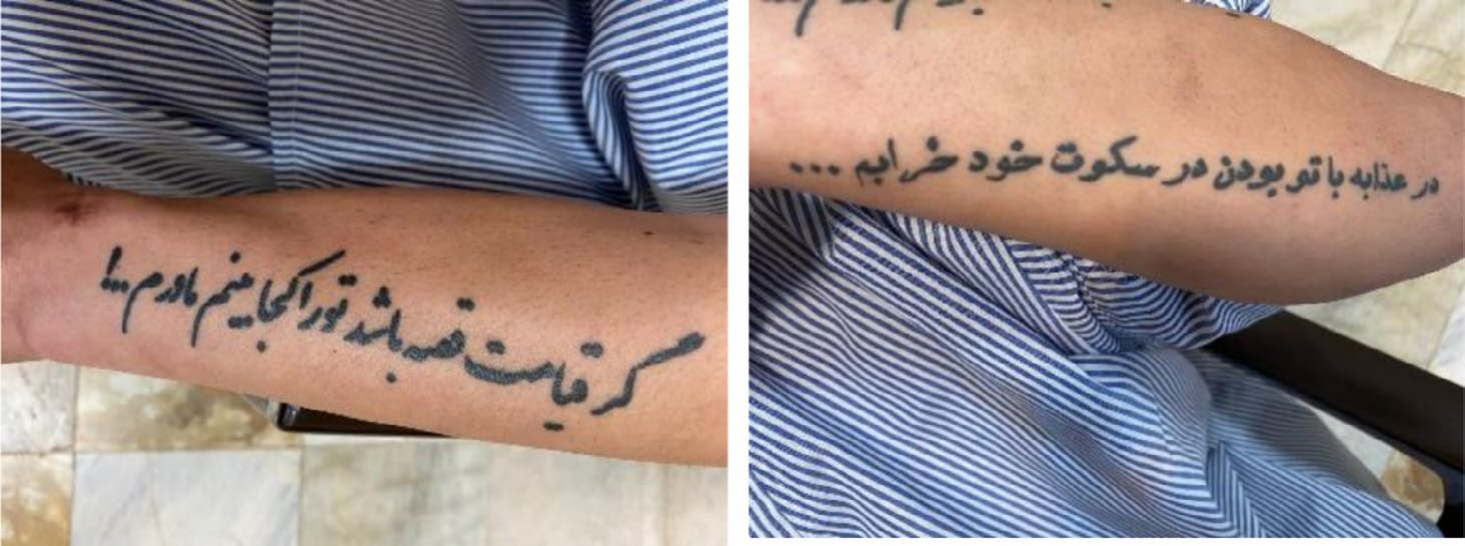
Tattoos on his left forearm. The picture on the right side: “Being with you is torment; in my silence, I am falling apart”. The picture on the left side: “If Judgment Day is only a tale, where will I find you, my mother?”

His personal history, including exhibited stuttering, aggression, and involvement in physical altercations, was notable. He voluntarily left school at the age of 15 years and worked in manual labor and construction but was unable to continue working after surgery. His mandatory military service was delayed by 2 years due to substance use and peer conflicts, although he eventually enlisted and subsequently deserted. Physical examination revealed multiple tattoos on his left forearm (Figure 1) and scars on his left arm, indicative of non‐suicidal self‐injury (NSSI). Although he had never attempted suicide, he frequently experienced thoughts of death and suicide.

He appeared cachectic and pale, with right‐sided claudication. He was cooperative and maintained good eye contact. His mood was depressed, and so was his affect. The patient exhibited low resilience and hyperalgesia, which made him highly sensitive to pain. He spoke in a low, monotone voice, although speech fluency was generally preserved. In the mental status examination, thought processes were logical and goal‐directed, with no evidence of formal thought disorder. He was alert and oriented to person, place, and time. Cognitive function was grossly intact with no significant impairment.

## 3 Methods (Differential Diagnosis, Investigations, and Treatment)

The patient was hospitalized in the psychiatric ward based on clinical interview and presence of depression, low resilience, poor therapeutic compliance, and unsupportive family and friends.

Furthermore, the patient was experiencing pain in the lower back and right knee secondary to mild claudication and leg length discrepancy resulting from previous avascular necrosis caused by chronic corticosteroid use and prior total hip arthroplasty. Further investigations were conducted, including a review of his previous medical records, and analgesic therapy was initiated. Additionally, he received medication for depression and was treated for cessation of drug use.

The patient had been admitted to the orthopedic clinic about 1 year prior to the present admission, complaining of pain and claudication in the groin and right lower limb. Clinical evaluation had begun with history taking and physical examination, and the differential diagnosis included bone marrow edema syndrome, complex regional pain syndrome, inflammatory synovitis, neoplastic bone conditions, osteoarthritis, osteochondrosis, osteomyelitis, osteoporosis, rheumatoid arthritis, septic arthritis, and subchondral fractures.

**FIGURE 2:**
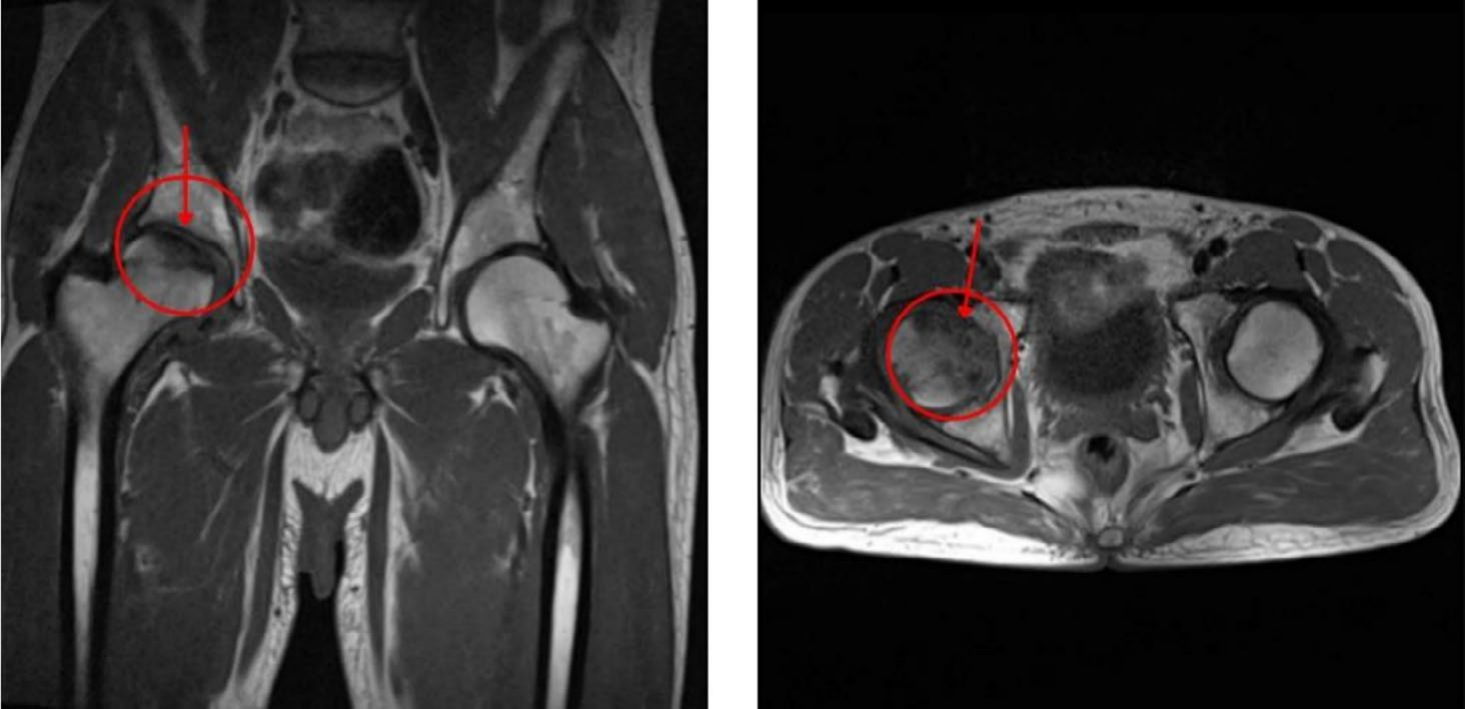
Pelvic MRI, evidence of subcortical crescent‐like signal abnormality in the right femoral head is noted due to osteonecrosis (Avascular necrosis (AVN)). Evidence of bone marrow edema at the right femoral head is seen. Mild effusion in the right hip joint is noted.

The history of alcohol use and long‐term corticosteroid misuse raised suspicion of AVN, and the first‐line imaging (X‐ray) demonstrated sclerosis, cystic changes, and a subchondral fracture. MRI, as the gold standard, confirmed AVN of the femoral head (Figure 2). The patient subsequently underwent THA of the right lower limb [5].

Postsurgical medication (aspirin, apixaban, and meloxicam) was prescribed to prevent deep vein thrombosis and surgical complications, and the patient was discharged from the orthopedic ward.

In the psychiatric ward, the patient received a consultation session about the consequences of inappropriate corticosteroid use, including the risk of AVN occurrence in other joints and worsening of depressive symptoms.

## 4 Conclusions and Results (Outcome and Follow‐Up)

The patient demonstrated a favorable response to treatment during hospitalization, and weakness and depressive symptoms diminished. Reassurance was provided, and the patient was encouraged to pursue a drug‐free lifestyle. The patient was subsequently discharged after partial recovery. He was advised to attend regular follow‐up sessions every 2 months at the clinic. He had two clinic visits with no evidence of drug or dexamethasone misuse. Furthermore, he reported gradual improvement in depression and reduction in weakness.

## 5 Discussion

This case underscores the complex psychiatric and physical consequences of chronic substance misuse, particularly the combined use of opioids and corticosteroids. Initially motivated by physical deterioration due to long‐term opioid use, manifested as weight loss, facial atrophy, and hyperpigmentation, the patient began using dexamethasone upon peer recommendation to counteract these changes. While dexamethasone temporarily improved his physical appearance and energy levels, its prolonged use resulted in significant psychiatric and orthopedic complications.

Dexamethasone, a synthetic corticosteroid, may initially produce euphoria and improved energy levels, which can reinforce continued misuse. However, prolonged corticosteroid use is associated with adverse psychiatric effects, such as mood lability, anxiety, and major depressive episodes [6].

In addition to psychiatric sequelae, chronic corticosteroid use can result in orthopedic complications, such as avascular necrosis (AVN) of the femoral head. The patient developed AVN of the femoral head, a serious and well‐documented complication associated with dexamethasone misuse.

AVN, also known as osteonecrosis, is a condition characterized by the disruption of blood supply to the bony tissue, leading to ischemia, cellular death, and eventual joint destruction [5]. It typically emerges in young adults and negatively affects the most active years of life [7].

AVN commonly involves weight‐bearing joints including the femoral head, and knee [8]. The femoral head is particularly susceptible due to its limited collateral blood supply and high weight‐bearing function [9].

This disease initially presents with progressive and localized joint pain, followed by a limited range of motion in the affected joint, indicating progressive destruction of bone integrity. Joint stiffness, often reported as difficulty initiating movement and morning stiffness, and muscle atrophy further compromise joint function and stability [8].

It may result from traumatic events, such as femoral head fractures and hip dislocations, or from non‐traumatic causes, among which corticosteroid use is the most common, followed by chronic alcohol abuse. Corticosteroids contribute to AVN through several mechanisms. These include adipocyte hypertrophy and proliferation, which increase bone marrow fat and intraosseous pressure, compromising vascular perfusion [2].

Fat embolism is another consequence of adipocyte hypertrophy, which can disrupt the microvascular circulation and impair blood flow [10]. In addition, the induction of apoptosis in bone microvascular endothelial cells (BMECs) and endothelial progenitor cells (EPCs) leads to vascular endothelial dysfunction and ischemia, which is accepted as a major mechanism in the pathogenesis of osteonecrosis [11]. Additionally, corticosteroids enhance thrombosis risk by altering fibrinolytic pathways and inducing vasoconstriction via endothelin‐1 and catecholamine release [12].

Another major pathological mechanism of AVN is through dysregulation of osteogenesis. Corticosteroids induce changes in mesenchymal stem cells (MSCs) and alter the expression of PPARγ in bone marrow mesenchymal stem cells (BMSCs), which are responsible for bone formation. These changes lead to adipogenic differentiation instead of efficient osteogenesis. Furthermore, dysregulation of pathways such as the OPG/RANK/RANKL system and the Wnt/β‐catenin signaling pathway is a key step in the occurrence of osteonecrosis and impaired bone integrity [11].

Moreover, advanced research in proteomics and genomics has provided further insights into the mechanisms, suggesting that proteomic investigations reveal widespread changes in protein expression, including immunosuppression, apoptosis, matrix metalloproteinase activity, and altered cation transport, while alterations in COL5A2 and serum amyloid A indicate reduced osteogenic capacity. Genomic analyses have identified susceptibility and risk‐associated genes, including MDR1, ABCB1, CYP450, MMP9, and MMP14 [11].

The diagnosis of AVN relies on imaging studies. While X‐rays may be used initially, MRI is the most sensitive and specific tool for diagnosing early‐stage AVN. Moreover, CT‐Scan plays a crucial role by contributing anatomical details to provide the best clinical management [8].

Prompt diagnosis and treatment, ranging from conservative therapies to surgical intervention, most efficiently THA, are vital to prevent irreversible joint destruction and permanent disability [13].

## 6 Conclusion

This case described a clear aspect of chronic and unsupervised dexamethasone use, which led to the development of depression, AVN, and lifelong disability due to chronic pain and claudication, affecting all aspects of a person's life, including social and professional standing. It also placed a significant financial burden on the family and society.

All of this emphasized the importance of corticosteroid screening in people with chronic opium use, particularly in those showing obvious signs of depression and drug‐induced deterioration in appearance.

## Author Contributions

**Reza Bidaki:** data curation, supervision, writing – review and editing. **Asma Khosravi Najafabadi:** investigation, writing – original draft, writing – review and editing. **Farzaneh Hasani:** data curation.

## Funding

The authors have nothing to report.

## Ethics Statement

The human studies were approved by the Ethics Committee of Shahid Sadoughi University of Medical Sciences, Yazd, Iran (Approval code: IR.SSU.MEDICINE.REC.1404.071). The local legislation and institutional requirements conducted the analyses.

## Consent

The participants provided their written informed consent to participate in this study. The patient provided written informed consent for the publication of any potentially identifiable images or data included in this article.

## Conflicts of Interest

The authors declare no conflicts of interest.

## Data Availability

All relevant information is provided within this article additional details may be available by corresponding author upon reasonable request due to ethical considerations.
